# Body Height of Children with Bronchial Asthma of Various Severities

**DOI:** 10.1155/2017/8761404

**Published:** 2017-07-26

**Authors:** Tatiana I. Eliseeva, Natalia A. Geppe, Elena V. Tush, Olga V. Khaletskaya, Ivan I. Balabolkin, Vilya A. Bulgakova, Nailya I. Kubysheva, Stanislav K. Ignatov

**Affiliations:** ^1^Department of Hospital Pediatrics, Nizhny Novgorod State Medical Academy, 10/1 Minin and Pozharsky Square, Nizhny Novgorod 603005, Russia; ^2^Department of Children's Diseases, I.M. Sechenov First Moscow State Medical University, 8/2 Trubetskaya St., Moscow 119991, Russia; ^3^Department of Pulmonology and Allergology, Federal State Autonomous Institution “National Scientific and Practical Center of Children's Health” of the Ministry of Health of the Russian Federation, Lomonosovsky Prospekt 2, Build. 1, Moscow 119991, Russia; ^4^Research Laboratory “Medical Informatics”, Higher School of Information Technologies and Information Systems, Kazan Federal University, 18 Kremlyovskaya St., Kazan 420008, Russia; ^5^Department of Chemistry, Lobachevsky State University of Nizhny Novgorod, 23 Prospekt Gagarina, Nizhny Novgorod 603950, Russia

## Abstract

Influence of bronchial asthma (BA) severity on physical development in children patients was evaluated in comparison with healthy population.* Materials and Methods*. 1042 children and adolescents (768 boys) with atopic BA were evaluated. All children underwent standard examination in a clinical setting, including anthropometry. The control group included 875 healthy children of a comparable age (423 boys).* Results*. The fraction of patients with the normal, lower, and increased height among the whole group of patients with BA is close to the corresponding values in the healthy population (*χ*^2^ = 3.32, *p* = 0.65). The fraction of BA patients with the reduced physical development is increased monotonically and significantly when the BA severity increases: healthy group, 8.2% (72/875), BA intermittent, 4.2% (6/144), BA mild persistent 9% (47/520), BA moderate persistent, 11.7% (36/308), and BA severe persistent, 24.3% (17/70) (*χ*^2^ = 45.6, *p* = 0,0009).* Conclusion*. The fraction of the children with the reduced height is increased monotonically and significantly in the groups of increasing BA severities. At the same time, the fraction of such children in groups of intermittent and mild persistent BA practically does not differ from the conditionally healthy peers.

## 1. Introduction

During last years, the connection between the physical development, nutritive status, and the disease course features in the patients with bronchial asthma (BA) attracts close attention [1–3]. In particular, the possible deceleration of the physical development in children and teenagers with BA, namely, the deceleration of their body growth as the leading parameter of physical development, is discussed [4, 5].

Several studies connected the negative impact of BA on children physical development to nutritional features (eliminative diets at a food sensitization) and the pharmaceuticals used in the BA therapy [6, 7]. It was suggested that children with BA having night symptoms can have disturbances of night secretion of somatotropic hormone. It, in turn, can cause their growth inhibition. At present, this hypothesis is not commonly accepted because no abnormalities in the urine excretion of somatotropic hormone were found in the BA patients [8].

Another factor potentially capable of growth inhibition in children and teenagers with BA is a glucocorticosteroid therapy [9]. The inhalation glucocorticosteroids (IGCS), which are in use for a long time as a basic anti-inflammatory BA treatment, is capable of influencing many key factors involved in the children body development: secretion and effect of somatotropic hormone, effect of an insulin-like growth factor, collagen synthesis, and production by adrenal glands of androgens [10, 11].

It should be noted that, at present, the studies about the correlation between physical development and BA are not numerous, and opinions about the influence of BA on the children body height are rather ambiguous. In the literature, the questions of influence of the IGCS therapy on the growth of children with asthma are mostly discussed, whereas the data on the prevalence of deviations in physical development in children with BA in comparison with population indexes are virtually absent.

In the current paper, we perform a comparative study of the body height deviations in the groups of Russian city children and teenagers with BA of various severities in comparison with the control group of conditionally healthy children of the same age. The main goal of the study is the elucidation of the abundance in the deviations of body height in children and teenagers with BA and establishing the correlation between these deviations and the severity of asthma.

During this study, we use the reference data recommended by the World Health Organization (WHO) for the assessment of the physical development in school-aged children and adolescents taking into account the gender and age [12]. It should be noted that various regional standards are frequently proposed in a literature in order to account the regional deviations in local populations [13]. On the other hand, the use of unified standards of WHO allows obtaining the standardized data in the conditions of escalating migration [12].

## 2. Materials and Methods

We performed a retrospective analysis of medical histories data on patients with atopic BA aged 5 (61 months) to 17 years (215 months), mean age 10.9 ± 3.7 years (134.7 ± 45.3 months), which were observed in the 1st Children City Clinical Hospital, Nizhny Novgorod, Russia, during the period from 2008 to 2015. The study included in total 1042 children and adolescents with atopic BA, including 768 boys and 274 girls (Table 1). All the children underwent standard examination in a clinical setting, including anthropometry. The diagnosis of BA and the disease severity were established by an attending doctor in accordance with recommendations valid at that period of time (GINA 2005–2015) [14]. Mild intermittent BA was diagnosed in 144 patients and mild persistent BA in 520 patients, moderately severe BA was found in 308 children, and 70 patients had severe BA (see Table 1).

The control group consisted of 875 healthy children (423 boys and 452 girls) of a comparable age from Nizhny Novgorod staying in the dispensary observation.

The study is retrospective; it was executed on the basis of the analysis of the data reflected in case histories of patients with BA and cards of dispensary survey of healthy children. It was approved by the Ethical Committee of the Sechenov 1st Moscow Medical University, Moscow, Russia (protocol number 05-11 of 19.05.2011).

The assessment of the body height was carried out taking into account the gender and age of patients on the basis of *Z*-score tables according to WHO recommendations [11]. Compliance of the children body height to median values of body height in the range of units from −1 to +1 on a scale of *Z*-score was considered as a normal physical development and in the range from +1 up to +2 as increased physical development; excess of body height of the patient more than by 2 units on *Z*-score scale was considered as high-tallness. Analogously, the body height in the range from −1 to −2 was considered as the decreased physical development and *Z*-score less than −2 as low-tallness.

### 2.1. Statistical Data Processing

The data are presented in absolute and relative units, as *M* ± *m*, where *M* is arithmetic mean, *m* is standard deviation, and the confidence interval of 95% is reported in some cases. The standard methods of ANOVA along with the statistical criteria *χ*^2^, Student's *t*-test, and Kruskal–Wallis test were used for analysis. Statistical calculations were carried out using Statgraphics Plus v.5 software. The differences were considered statistically significant at *p* < 0.05.

## 3. Results and Discussion

More than one-half of examined children with BA, 63.5% (662/1042), had normal physical development (see Table 2), including 63.0% of boys (484/768) and 65.0% (178/274) of girls. Decreased body height is revealed in 10.1% (106/1042) of patients. Among them, 1.6% (17/1042) had low-tallness (height lower than 2*Z*). Boys have decreased body height in 9.2% cases (71/768); the low-tallness (lower than 2*Z*) was revealed in 1.4% cases (11/768). Girls had decreased level of physical development in 12.7% patients (35/274), with significant low-tallness in 2.2% (6/274) of them.

A remarkable fraction of patients with BA had an increased physical development. In the whole group of BA patients, the increased height was in 26.2% (274/1042) patients; in 6.0% (63/1042) of cases the increase was higher than 2*Z*. Among boys the increased physical development took place in 27.8% (213/768), higher than 2*Z*, in 6.9% (53/768) of the cases. Among girls the increased physical development is revealed in 22.3% (61/274) patients; body height excess more than 2*Z* took place at 3.7% (10/274) children.

The ratios of patients with the normal, lower, and increased physical development in the whole group of patients with BA are quite similar to the population of healthy children (no statistical differences, *χ*^2^ = 3.32, *p* = 0.65).

**Figure 1:**
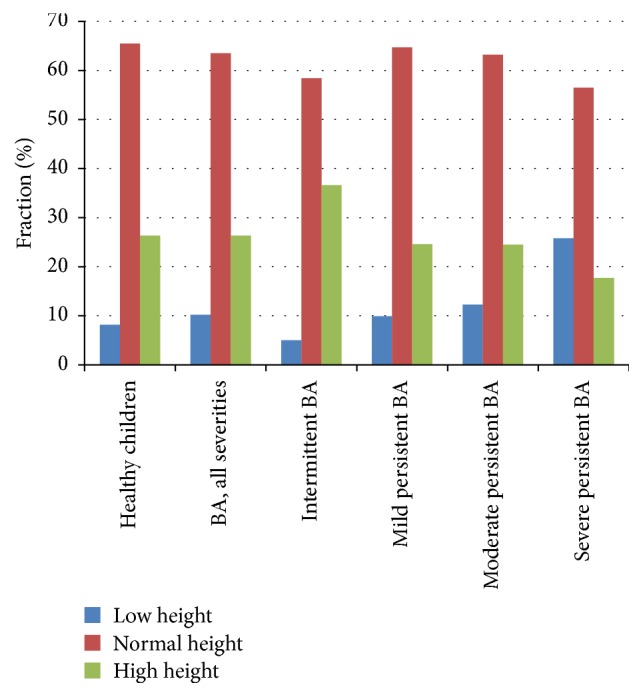
Physical development of children with different BA severity.

However, the analysis of physical development in the groups of patients with various BA severities demonstrates the clear statistical distinctions in the groups of children having the reduced physical development (see Table 2 and Figure 1). In the group of intermittent BA there was only 4.2% (6/144) of children with low height, and this quantity is increased monotonically when BA severity increased: in the group of mild persistent BA, 9.0% (47/520), moderate persistent BA, 11.7% (36/308), and severe persistent BA, 24.3% (17/70). Along with this trend, the fraction of the children having the increased physical development monotonically decreases: intermittent BA, 37.5% (54/144), mild persistent BA, 25.8% (134/520), moderate persistent BA, 24.0% (74/308), and severe persistent BA, 17.1% (12/70). Distinctions are statistically significant both with the whole group of patients (*χ*^2^ = 32.84, *p* ≤ 0.0001) and with separate groups of boys (*χ*^2^ = 33,91, *p* ≤ 0,0001) and girls (*χ*^2^ = 10,37, *p* = 0,0200).

The revealed regularities are also confirmed by the analysis of the relative index of body height RI (measured body height divided by the recommended median value of body height for this gender and age) and standard deviation score SDS (difference between the measured body height and its recommended median value divided by the recommended standard deviation); see Tables 3 and 4. The analysis of these parameters demonstrates that the severity of BA is associated with the monotonous and statistically significant decrease in the mean body height values in patients with BA.

The significant distinctions of physical development between the whole group of patients with BA and healthy children during various age periods are not observed, as it can be concluded from the comparison of the RI and SDS values for the whole group of BA patients and control group (*F* = 1.74, *p* = 0.19); see Table 5.

## 4. Discussion

The physical development of children with BA was similar to the population of healthy children. The gender- and age-specific distinctions of physical development between patients with BA and healthy children were also not revealed.

However, in spite of the fact that more than one-half of the examined children with BA of all severities had normal physical development, the fraction of children with the reduced physical development increases monotonically in the groups with increased BA severity. In the group with severe persistent BA, 24.3% of patients had low physical development. Statistically significant distinctions of physical development were found among patients of different severities as well as for the whole group of patients (*χ*^2^ = 32.84, *p* = 0.000). This took place separately both for boys (*χ*^2^ = 33.91, *p* = 0.000) and for girls (*χ*^2^ = 10.37, *p* = 0.020).

Interestingly, the group of intermittent BA has even larger fraction of children with high growth (37.5%, 54/144) than in the control group (26.3%, 230/875). This feature cannot be explained by the effect of cromones as it was proposed earlier because cromone therapy was not used in any observed case [15]. It is also obvious that the patients with the compensated disease who are not receiving the glucocorticosteroids potentially influencing body height, or receiving them in low doses, should not differ from the common population. The fact that this group has higher body height than the healthy group, undoubtedly, demands more attention. In our opinion, the fact should also be taken into account that the fraction of asthenic girls is especially high in this group.

At the same time, the group with severe persistent BA significantly differs from the control children and the children with mild and moderate persistent BA by the increased fraction of patients with reduced physical development, 24.3% (17/70), of patients with severe BA against 8.2% (72/875) in a control group,as well as by the decreased fraction of children with the increased physical development, 17.1% (12/70) against 26.3% (230/875). At the moment, we cannot conclude whether this fact is a consequence of basic disease, or a therapy. Undoubtedly, special attention should be paid to these observations.

It is known that the children body height is affected by the sport exercises. Namely, the synthesis of somatotropin was observed as the response for the dosed physical training which positively influences the children body height [16]. This fact can affect the results obtained in the present study. Unfortunately, the sport training was not systematically described in the medical histories studied by us. It is very probably that the patients with severe BA do not get sports like others. In principle, this fact can explain the lower body height values in this group. However, this does not explain the results for the group with light intermittent BA where the measured values were higher than in the control group.

In the recent study, it was found by us that children with different BA severity are also distinguished by their nutritive status estimated by the body mass index (BMI). Probably, the deviations in body height and body mass have the common nature and pathophysiological mechanism [3]. As it was demonstrated earlier, BMI deviations have the close connection to the metabolomic profiles of the BA patients and correspond to the different asthma phenotypes [17]. Thus, it is quite probably that the metabolomic studies can also be useful in the analysis of the height deviations in BA and can reveal new details in the pathophysiological mechanism of this disease.

## 5. Conclusion 

It was found that the fraction of children with the reduced physical development increases monotonically and significantly in the groups of patients with increasing BA severity whereas the fraction of children with the increased physical development decreases in the same direction. At the same time, the fraction of children with reduced physical development in the group of mild persistent BA does not differ significantly from the control group of conditionally healthy peers and this fraction of children with the increased height in the group of intermittent BA is even higher than in the control group. These facts have no clear explanations on the basis of known effects of modern medicines and require additional investigations. They should be taken into account during the planning of further BA studies.

## Figures and Tables

**Table 1:** Clinical profile of subjects.

Characteristic	Number of patients	Gender (boys/girls)
Healthy	875	423/452
5-6 years	206	102/104
7–9 years	193	102/91
10–12 years	198	89/108
13-14 years	125	62/63
15–17 years	154	68/86
BA	1042	768/274
5-6 years	189	111/78
7–9 years	208	136/71
10–12 years	241	186/55
13-14 years	164	137/27
15–17 years	241	198/43
Degree of bronchial asthma severity:		
Intermittent	144	115/29
Mild persistent	520	385/135
Moderate persistent	308	217/91
Severe persistent	70	51/19

**Table 2:** Distribution of patients from BA various weight using the WHO *Z*-score system (absolute number/fraction in %). Cross-tabulation demonstrates dependency between *Z*-score and asthma severity (*χ*^2^ = 45.6, *p* = 0.0009).

Degree of bronchial asthma severity:	Lower than −2	From −2 to −1	From −1 to 1	From 1 to 2	From 2 to 3	Higher than 3	Total
Total with BA	17/1.6	89/8.5	662/63.5	211/20.3	56/5.3	7/0.7	1042/100
Intermittent	0/0	6/4.2	84/58.3	38/26.4	14/9.7	2/1.4	144/100
Mild persistent	9/1.7	38/7.3	339/65.2	105/20.2	26/5.0	3/0.6	520/100
Moderate persistent	7/2.3	29/9.4	198/64.3	56/18.2	16/5.2	2/0.7	308/100
Severe persistent	1/1.4	16/22.9	41/58.6	12/17.1	0/0	0/0	70/100
Healthy control group	9/1.0	63/7.2	573/65.5	180/20.6	42/4.8	8/0.9	875/100

**Table 3:** Relative index (RI) of body height in children with various severity of BA in comparison with healthy children.

Group	Healthy	BA severity				Statistics (ANOVA)
		Intermittent	Mild persistent	Moderate persistent	Severe persistent	
All	1.018 ± 0.002	1.032 ± 0.004	1.015 ± 0.002	1.010 ± 0.003	0.998 ± 0.006	*F* = 8.0, *p* ≤ 0.0001
Boys	1.018 ± 0.002	1.030 ± 0.004	1.019 ± 0.002	1.013 ± 0.003	0.995 ± 0.007	*F* = 5.2, *p* = 0.0004
Girls	1.018 ± 0.002	1.42 ± 0.009	1.006 ± 0.004	1.005 ± 0.005	1.005 ± 0.010	*F* = 5.3, *p* = 0.0003

**Table 4:** Standard deviation score (SDS) at children with various severity of BA in comparison with healthy children.

Group	Healthy	BA severity				Statistics (ANOVA, Fisher test)
		Intermittent	Mild persistent	Moderate persistent	Severe persistent	
All	0.39 ± 0.35	0.71 ± 0.09	0.33 ± 0.05	0.24 ± 0.060	–0.05 ± 0.13	*F* = 8.0, *p* ≤ 0.0001
Boys	0.40 ± 0.05	0.65 ± 0.10	0.41 ± 0.05	0.29 ± 0.07	–0.11 ± 0.15	*F* = 5.2, *p* = 0.0004
Girls	0.39 ± 0.05	0.93 ± 0.19	0.12 ± 0.09	0.12 ± 0.11	0.11 ± 0.24	*F* = 5.3, *p* = 0.0003

**Table 5:** Relative index (RI) of body height in the BA patients of different age and sex in comparison with healthy children.

Age	Gender	Healthy	BA patients	Statistics
5-6	Boys	1.008 ± 0.002	1.013 ± 0.002	KW test = 0.9, *p* = 0.34
	Girls	1.060 ± 0.002	1.007 ± 0.002	KW test = 0.0005, *p* = 0.98
	Total	1.007 ± 0.003	1.011 ± 0.003	KW test = 0.7, *p* = 0.4

7–9	Boys	1.010 ± 0.005	1.018 ± 0.004	KW test = 1.1, *p* = 0.3
	Girls	1.024 ± 0.005	1.012 ± 0.006	KW test = 1.31, *p* = 0.25
	Total	1.016 ± 0.004	1.016 ± 0.003	KW test = 0.003, *p* = 0.9

10–12	Boys	1.031 ± 0.005	1.018 ± 0.004	KW test = 3.46, *p* = 0.06
	Girls	1.025 ± 0.005	1.006 ± 0.007	KW test = 3.3, *p* = 0.07
	Total	1.028 ± 0.002	1.015 ± 0.003	KW test = 5.4, *p* = 0.02

13-14	Boys	1.032 ± 0.006	1.024 ± 0.004	KW test = 0.5, *p* = 0.5
	Girls	1.010 ± 0.005	0.999 ± 0.008	KW test = 1.1, *p* = 0.3
	Total	1.021 ± 0.004	1.020 ± 0.004	KW test = 0.05, *p* = 0.8

15–17	Boys	1.017 ± 0.005	1.013 ± 0.003	KW test = 1.1, *p* = 0.3
	Girls	1.021 ± 0.004	1.014 ± 0.006	KW test = 0.7, *p* = 0.4
	Total	1.019 ± 0.003	1.013 ± 0.003	KW test = 2.3, *p* = 0.13
